# Magnetic Poly(VPA-co-EGDMA)-TiO_2_ Polymeric Microspheres as Supports for Acetylcholinesterase Immobilization: Kinetic Behavior, Stability, and Reusability

**DOI:** 10.3390/polym18161999

**Published:** 2026-08-17

**Authors:** Şahin Özel

**Affiliations:** Department of Chemistry, Faculty of Science and Arts, Bursa Uludağ University, Bursa 16059, Turkey; sahinozel@uludag.edu.tr

**Keywords:** enzyme stability and kinetics, multifunctional polymeric microspheres, acetylcholinesterase immobilization, reusability

## Abstract

Magnetic poly(vinylphosphonic acid-co-ethylene glycol dimethacrylate)-TiO_2_ [m-poly(VPA-co-EGDMA)-TiO_2_] microspheres were synthesized by suspension polymerization and evaluated as supports for direct acetylcholinesterase (AChE) immobilization. The microspheres were characterized by SEM, FT-IR, BET, XRD, XPS, VSM, and zeta potential analyses and exhibited porous morphology, magnetic responsiveness, a specific surface area of 195 m^2^ g^−1^, and a swelling ratio of 40.19%. AChE was immobilized mainly through physical adsorption and multipoint non-covalent enzyme–support interactions. The optimum pH was pH 10 for both free and immobilized AChE. Under the tested assay conditions, immobilized AChE showed apparent kinetic parameters of Km = 0.028 mM and Vmax = 0.132 EU.mL^−1^. Immobilized AChE retained 58.2% of its initial activity after 60 days at 4 °C and 24% after 20 reuse cycles. Overall, the microspheres offer a magnetically recoverable support for AChE immobilization, although further optimization is required to improve kinetic performance and long-term operational stability.

## 1. Introduction

The immobilization of enzymes on solid supports has attracted significant attention due to the improved stability, reusability, and operational durability of immobilized enzymes compared with free enzymes [1,2,3]. Immobilized enzymes exhibit enhanced stability against environmental conditions, improved operational durability, and reusability, which contribute to cost-effective and sustainable applications [4,5]. In addition, immobilization enables easier separation of enzymes from reaction media and facilitates the production of high-purity products, particularly in medical and analytical fields [6,7]. In recent years, increasing environmental and health concerns related to the extensive use of pesticides have driven the development of sensitive and reliable detection methods. Among these, enzyme-based biosensors have emerged as powerful tools, with acetylcholinesterase (AChE) being one of the most widely used enzymes. AChE plays a crucial role in detecting organophosphate and carbamate pesticides due to its sensitivity to inhibitors, which irreversibly bind to the catalytic serine residue in its active site, leading to inhibition of neurotransmission. This inhibition mechanism forms the basis of AChE-based biosensing systems [8,9,10,11]. To enhance enzyme performance in such applications, various immobilization strategies have been developed using organic and inorganic support materials [12]. Common immobilization techniques include physical adsorption, entrapment, and covalent attachment. Among these, covalent immobilization using bifunctional cross-linking agents such as glutaraldehyde is particularly advantageous due to its ability to provide strong and stable enzyme–support interactions while minimizing enzyme leakage [13,14]. Magnetic nanoparticles, especially Fe_3_O_4_-based systems, have been widely investigated as support materials for enzyme immobilization because of their magnetic responsiveness, tunable surface functionality, and ease of separation under an external magnetic field [15]. These properties can facilitate recovery and reuse of immobilized enzymes, although the actual immobilization performance depends strongly on the support structure, surface chemistry, and immobilization method [16]. Furthermore, surface functionalization of Fe_3_O_4_ nanoparticles enhances enzyme stability and catalytic efficiency, making them suitable platforms for advanced biotechnological systems [17,18]. In parallel, polymer-based porous materials have gained attention as multifunctional platforms due to their tunable structure [19], high surface area, and chemical versatility [20,21,22,23]. In this context, magnetic cross-linked microspheres represent a promising class of materials combining magnetic properties, porosity, and functional groups suitable for various applications. These microspheres exhibit high surface area, hydrophilicity, and structural stability, which are advantageous for adsorption and catalytic processes. Although various support materials have been investigated for AChE immobilization, the development of functional, recoverable, and porous supports for enzyme attachment remains an important research area. AChE was selected as the model enzyme in this study because of its relevance to inhibition-based biosensing systems, particularly those used for organophosphate and carbamate pesticide detection. However, the present study does not aim to construct a complete biosensor or environmental monitoring platform. No pesticide inhibition assay, analytical calibration curve, selectivity test, detection limit determination, or real-sample analysis was performed. Therefore, biosensing and environmental monitoring are discussed only as potential future applications. The principal aim of this study was to prepare magnetic m-poly(VPA-co-EGDMA)-TiO_2_ porous microspheres and evaluate their suitability as supports for direct AChE immobilization. The synthesis approach was adapted from previously reported polymeric microsphere preparation methods. Therefore, the novelty of the present work is not claimed solely on the basis of the synthesis of the support material. Rather, the novelty lies in the application and evaluation of magnetic m-poly(VPA-co-EGDMA)-TiO_2_ porous microspheres as cross-linker-free supports for direct AChE immobilization.

This microsphere platform combines VPA-derived phosphonic acid functionality, an EGDMA-based cross-linked polymeric network, TiO_2_ incorporation, porous morphology, and magnetic responsiveness. These features may provide functional interaction sites and facilitate magnetic recovery of the immobilized enzyme system. The current evaluation focuses on the suitability of the prepared microspheres for AChE immobilization in terms of apparent kinetic behavior, pH-dependent activity, thermal stability, storage stability, and reusability. Future studies should demonstrate practical applicability using model pesticide inhibition assays, analytical calibration, sensitivity, selectivity, detection limit, and real-sample validation.

## 2. Materials and Methods

### 2.1. Materials

Ethylene glycol dimethacrylate (EGDMA) was purchased from Merck (Darmstadt, Germany), while vinylphosphonic acid (VPA) and 2,2′-azobisisobutyronitrile (AIBN) were obtained from Fluka A.G. (Buchs, Switzerland). Poly(vinyl alcohol) (PVA; Mw: 100,000, 98% hydrolyzed), toluene, ethanol, nitric acid (HNO_3_), TiO_2_ (P25 Aeroxide, Degussa, Düsseldorf, Germany), and benzoyl peroxide (BPO) were supplied by Sigma-Aldrich (St. Louis, MO, USA). Magnetite nanoparticles (Fe_3_O_4_; 20–50 nm) were procured from Merck (Darmstadt, Germany). Acetylcholinesterase (AChE), acetylthiocholine iodide (ATCh), monobasic sodium phosphate, and disodium phosphate were purchased from Sigma-Aldrich (St. Louis, MO, USA) and used as received. All chemicals were of analytical grade and used without further purification. Deionized water used in all experiments was purified using a Barnstead ROpure LPw reverse osmosis system followed by a Barnstead NANOpure purification unit (Thermo Fisher Scientific, Waltham, MA, USA).

### 2.2. Synthesis of Polymer Microspheres

[m-poly(VPA-co-EGDMA)-TiO_2_] microspheres were synthesized via suspension polymerization using an aqueous dispersion phase and an organic phase [24]. For the dispersion phase, 0.2 g of PVA was dissolved in 50 mL of distilled water under heating and magnetic stirring until complete dissolution. The organic phase was prepared by dissolving 0.2 g of benzoyl peroxide (BPO) in 10 mL of toluene, followed by the addition of 5.4 mL of EGDMA (30 mmol) and 3.2 mL of VPA (30 mmol) to obtain a homogeneous mixture. The polymerization reaction was carried out in a Pyrex cylindrical batch reactor equipped with an electromantle heater at 65 °C. The dispersion phase was first introduced into the reactor, followed by the addition of 0.5 g of Fe_3_O_4_ nanoparticles and 0.5 g of TiO_2_ nanoparticles. The organic phase was then added under continuous magnetic stirring. Additionally, 100 mg of AIBN and 3 mL of VPA were incorporated into the organic phase and dispersed into the aqueous phase. Polymerization was conducted at 65 °C for 24 h at a stirring speed of 800 rpm. After completion of the reaction, the dispersion medium was decanted, and the obtained microspheres were washed with an ethanol–water mixture to remove unreacted monomers and residual solvents. The purified microspheres were then dried in a vacuum oven at 70 °C for 48 h.

### 2.3. Characterization

The synthesized magnetic polymeric microspheres were characterized using SEM–EDX, FT-IR, BET/BJH, XRD, XPS, VSM, zeta potential, and TGA/DTA analyses. The morphology and surface characteristics of the microspheres before and after AChE immobilization were examined by scanning electron microscopy (SEM) using a ZEISS EVO 40 scanning electron microscope equipped with an energy-dispersive X-ray spectroscopy (EDX) detector (Carl Zeiss Microscopy GmbH, Jena, Germany). Prior to SEM analysis, dried microsphere samples were mounted on aluminum stubs using conductive carbon tape and coated with a thin gold layer under vacuum to improve electrical conductivity. SEM images were recorded at different magnifications under high-vacuum conditions. SEM–EDX analysis was performed on selected regions of the samples to determine the elemental composition of the microspheres before and after enzyme immobilization. Fourier-transform infrared spectroscopy (FT-IR) was used to identify the characteristic functional groups of the synthesized microspheres and to evaluate spectral changes after AChE immobilization. FT-IR spectra were recorded using a PerkinElmer Spectrum Two FT-IR spectrometer (PerkinElmer, Waltham, MA, USA) in the wavenumber range of 4000–400 cm^−1^. Before measurement, bare and AChE-immobilized microsphere samples were dried to remove residual moisture and analyzed under identical measurement conditions.

The specific surface area and pore characteristics of the magnetic polymeric microspheres were determined by nitrogen adsorption–desorption analysis using a Micromeritics ASAP 2020 surface area and porosity analyzer (Micromeritics Instrument Corporation, Norcross, GA, USA). Before analysis, dried microsphere samples were degassed under vacuum at 80 °C for 12 h to remove adsorbed moisture and volatile impurities. The specific surface area was calculated using the Brunauer–Emmett–Teller (BET) method, while pore size distribution was evaluated using the Barrett–Joyner–Halenda (BJH) method.

The crystalline structure of the synthesized magnetic polymeric microspheres was analyzed by X-ray diffraction (XRD) using a Rigaku D/Max-2200 X-ray diffractometer (Rigaku Corporation, Tokyo, Japan). Dried microsphere samples were gently ground and placed onto the sample holder before measurement. XRD patterns were recorded over a 2θ range of 10–80° using Cu Kα radiation. The obtained diffraction peaks were used to identify the crystalline phases of TiO_2_ and Fe_3_O_4_ incorporated into the polymeric microsphere structure.

X-ray photoelectron spectroscopy (XPS) was performed to determine the surface elemental composition and chemical states of the synthesized microspheres using a Thermo Scientific K-Alpha XPS system (Thermo Fisher Scientific, Waltham, MA, USA). Prior to analysis, dried microsphere samples were fixed onto conductive sample holders. Survey spectra were recorded to identify the main surface elements, including C, O, P, Ti, and Fe. The obtained XPS data were used to confirm the formation of the m-poly(VPA-co-EGDMA)-TiO_2_ magnetic polymeric structure.

The magnetic properties of the synthesized microspheres were evaluated using vibrating sample magnetometry (VSM) with a Lake Shore 7404 vibrating sample magnetometer (Lake Shore Cryotronics, Inc., Westerville, OH, USA). Dried microsphere samples were placed into the sample holder and analyzed at room temperature. Magnetization curves were recorded as a function of the applied magnetic field, and the saturation magnetization value was determined from the obtained magnetization curve.

The surface charge behavior of the magnetic polymeric microspheres was investigated by zeta potential measurements using a Malvern Zetasizer Nano ZS analyzer (Malvern Instruments Ltd., Malvern, UK). For measurement, microsphere samples were dispersed in deionized water and briefly sonicated to obtain a homogeneous suspension. The pH of the suspensions was adjusted to the desired values using dilute HCl or NaOH solutions. Zeta potential values were recorded at different pH values to determine the surface charge behavior and isoelectric point of the microspheres.

Thermal properties of the synthesized microspheres were evaluated by thermogravimetric and differential thermal analysis (TGA/DTA) using a PerkinElmer STA 6000 thermal analyzer (PerkinElmer, Waltham, MA, USA). Dried microsphere samples were placed in an alumina pan and heated from room temperature to 600 °C under nitrogen atmosphere at a heating rate of 10 °C min^−1^. The thermal degradation behavior and inorganic residue content of the microspheres were determined from the obtained thermograms.

### 2.4. Enzyme Activity Assay

The enzymatic activity of free and immobilized acetylcholinesterase (AChE) was determined using the modified Ellman spectrophotometric method [25,26]. Briefly, an appropriate amount of AChE-loaded magnetic polymeric microspheres was dispersed in 4 mL of Tris–HCl buffer solution (0.1 M, pH 8.0) and gently stirred for 5 min to obtain a homogeneous suspension. Then, 50 μL of acetylthiocholine iodide (ATChI, 10 mM) and 50 μL of DTNB solution (5,5′-dithiobis-(2-nitrobenzoic acid), 10 mM) were added to the reaction mixture. In the Ellman method, thiocholine produced by the enzymatic hydrolysis of ATChI reacts with DTNB, resulting in the formation of the yellow-colored 5-thio-2-nitrobenzoate (TNB) anion. The absorbance of TNB was measured spectrophotometrically at 412 nm to determine AChE activity. The activity of free AChE was measured under the same experimental conditions for comparison. In addition, control measurements were performed using non-enzyme-loaded magnetic polymeric microspheres to evaluate possible background absorbance from the support material.

All measurements were performed in triplicate, and the results were expressed as mean ± standard deviation.

### 2.5. Protein Concentration Determination

Protein concentration was determined using the Bradford colorimetric protein assay. Briefly, Coomassie Brilliant Blue reagent was mixed with enzyme-containing samples, and the absorbance was measured at 595 nm after color development [27]. Bovine serum albumin (BSA) was used as the standard protein to construct the calibration curve. Protein concentration data were used only where protein-normalized activity expression was applicable. For the pH-dependent activity results, enzyme activity is expressed as AChE activity (EU.μg^−1^). These values should not be interpreted as newly recalculated protein-loading or activity-recovery data. This analytical method was included to clarify the protein quantification procedure. However, a complete protein mass balance including the initial enzyme solution, post-immobilization supernatant, and washing fractions was not performed in the present study. Therefore, protein-based enzyme loading, expressed as the amount of enzyme protein bound per unit mass of support, was not reported as an experimental result.

### 2.6. pH-Dependent Activity and Thermal Stability of AChE

The pH-dependent activity of free and immobilized acetylcholinesterase (AChE) was investigated over a pH range of 5–11 using the modified Ellman spectrophotometric method described in Section 2.4. Enzyme activity measurements were carried out directly in buffer solutions adjusted to the desired pH values. Briefly, free AChE solution and AChE-loaded magnetic polymeric microspheres were placed into separate reaction tubes containing buffers of different pH values. For immobilized AChE, 1 mg of AChE-loaded magnetic polymeric microspheres was used for each pH condition. The enzymatic reaction was initiated by the addition of acetylthiocholine iodide (ATChI) and DTNB. The pH-dependent activity experiments were performed at 25 °C using ATChI at a final substrate concentration of 1.0 mM, and the absorbance increase was monitored at 412 nm. Since the enzyme activity values in this analysis are presented as absolute enzyme activity, the results were expressed as AChE activity in EU.μg^−1^ rather than relative activity. The pH value at which the highest measured enzyme activity was observed was defined as the optimum pH under the tested assay conditions. This experiment was used to evaluate pH-dependent activity, not pH stability, because enzyme samples were not preincubated at different pH values followed by residual activity measurement under standard conditions. Accordingly, the results are discussed as pH-dependent activity data rather than pH stability data. All measurements were performed in triplicate, and the results were expressed as mean ± standard deviation. The thermal stability of free and immobilized AChE was evaluated by incubating enzyme samples at different temperatures ranging from 20 to 80 °C for 2 h. After thermal incubation, residual enzyme activity was measured under standard assay conditions using ATChI as the substrate and DTNB as the chromogenic reagent. The absorbance was recorded spectrophotometrically at 412 nm. Relative residual activity values were calculated by taking the highest measured residual activity as 100%. Since the enzyme samples were pre-incubated at different temperatures and then assayed under standard conditions, this experiment was used to evaluate thermal stability rather than the optimum catalytic reaction temperature. All measurements were performed in triplicate, and the results were expressed as mean ± standard deviation.

### 2.7. Immobilization of AChE onto Magnetic Polymeric Microspheres

Briefly, 10 mg of synthesized magnetic polymeric microspheres was suspended in 1 mL of phosphate buffer (50 mM, pH 7.6) and equilibrated at 25 °C under gentle shaking. Subsequently, 100 μL of AChE solution (activity: 0.5 EU.mL^−1^) prepared in phosphate buffer (50 mM, pH 7.6) was added to the microsphere suspension and incubated at 25 °C under gentle shaking to allow enzyme immobilization. After immobilization, the AChE-loaded magnetic polymeric microspheres were magnetically separated and washed three times with phosphate buffer (50 mM, pH 7.6) to remove unbound or weakly adsorbed enzyme. The immobilized enzyme was stored at 4 °C until further use. AChE was immobilized directly onto the synthesized magnetic polymeric microspheres without using any external cross-linking agent. The surface functionality of the microspheres originates mainly from phosphonic acid groups introduced by VPA, as well as hydroxyl-containing TiO_2_ and Fe_3_O_4_ components. These functional groups can interact with enzyme molecules through hydrogen bonding, electrostatic interactions, and possible coordination-type interactions. In particular, amino groups of lysine residues, carboxyl groups of aspartic and glutamic acid residues, and hydroxyl-containing amino acid residues in the AChE structure may contribute to enzyme–support interactions [7]. Therefore, AChE immobilization on the magnetic polymeric microspheres is considered to occur mainly through physical adsorption and multipoint non-covalent interactions. The immobilization performance was evaluated based on the decrease in enzyme activity measured in the supernatant after immobilization. The activity of the initial enzyme solution and the residual activity in the supernatant after immobilization were measured using the Ellman method [28]. The activity-based apparent immobilization value was calculated according to Equation (1) [29,30]. This value should be interpreted as an activity-based apparent immobilization parameter rather than a complete immobilization yield, activity recovery, or direct protein-based enzyme loading.

Activity-based apparent immobilization value (%) = [(A_0_ − A_s_)/A_0_] × 100 (1)

where A_0_ is the initial enzyme activity before immobilization and A_s_ is the residual enzyme activity measured in the supernatant after immobilization. Since this value was calculated only from activity measurements in the supernatant, it should be interpreted as an activity-based apparent immobilization parameter rather than a complete immobilization yield, activity recovery, or direct protein-based enzyme loading.

### 2.8. Storage Stability

The storage stability of free and immobilized acetylcholinesterase (AChE) was evaluated by storing enzyme samples at 4 °C for a period of 60 days. The enzyme solutions were prepared in buffer and kept under identical storage conditions throughout the experiment. At predetermined time intervals, aliquots of both free and immobilized enzyme samples were withdrawn, and their residual activities were determined using the modified Ellman spectrophotometric method with acetylthiocholine iodide (ATChI) as the substrate. The residual activity measurements were performed at 25 °C in 50 mM phosphate buffer (pH 7.6) using ATChI at a final substrate concentration of 1.0 mM and the same enzyme concentration/activity used in the initial assay. The enzyme activity measured at the initial time (day 0) was taken as 100%, and the remaining activity at each time point was expressed as a percentage of the initial activity. All measurements were carried out in triplicate, and the average values were reported.

### 2.9. Determination of Kinetic Parameters

The kinetic parameters of free and immobilized acetylcholinesterase (AChE) were determined using acetylthiocholine iodide (ATChI) as the substrate at varying concentrations. For kinetic analysis, ATChI solutions were prepared in phosphate buffer (50 mM, pH 7.6) at concentrations ranging from 0.01 to 1.0 mM. Enzyme activity was measured using the modified Ellman spectrophotometric method [25]. Briefly, the reaction mixture contained 100 μL of enzyme solution or immobilized enzyme suspension, 100 μL of DTNB solution (1.0 mM), 100 μL of ATChI solution at the desired concentration, and 700 μL of phosphate buffer (50 mM, pH 7.6), giving a final reaction volume of 1.0 mL. The reaction was carried out at 25 °C, and the absorbance increase was monitored at 412 nm for 5 min. Initial reaction rates were calculated from the linear portion of the absorbance–time curves. One enzyme unit was defined as the amount of enzyme catalyzing the formation of 1 μmol of product per minute under the assay conditions. Enzyme activity was calculated using the molar extinction coefficient of TNB at 412 nm, ε = 13,600 M^−1^ cm^−1^. The Michaelis–Menten kinetic parameters, namely Km and Vmax, were determined by fitting the experimental data to the Michaelis–Menten equation. All measurements were performed in triplicate, and the results were expressed as mean ± standard deviation. The reusability of immobilized AChE was evaluated through repeated catalytic cycles. After each cycle, the AChE-loaded magnetic polymeric microspheres were magnetically separated, washed with phosphate buffer solution (50 mM, pH 7.6), and reintroduced into a fresh substrate solution. The reaction cycles were conducted at 25 °C, and enzyme activity was measured after each cycle using the same modified Ellman assay. The residual activity after each cycle was calculated relative to the initial activity of the immobilized enzyme.

### 2.10. Determination of Magnetic Particle Volume Fraction and Swelling Ratio

The volumetric fraction of magnetic particles in the microspheres was calculated using Equation (2):

Magnetic particle volume fraction (%) = [(ρm − ρp)/(ρFe_3_O_4_ − ρp)] × 100 (2)

where ρm is the density of the magnetic microspheres, ρp is the density of the non-magnetic polymer matrix, and ρFe_3_O_4_ is the density of Fe_3_O_4_ particles. The swelling behavior of the microspheres was also investigated due to their porous and hydrophilic structure. The equilibrium swelling ratio was calculated using Equation (3):

Equilibrium swelling ratio = [(m_2_ − m_1_)/m_1_] × 100 (3)

where m_1_ and m_2_ represent the dry and swollen masses of the microspheres, respectively.

### 2.11. Statistical Analysis

Data analysis and graphical visualizations were performed using GraphPad Prism version 8 for Windows (GraphPad Software, La Jolla, CA, USA). All experimental measurements were performed in triplicate unless otherwise stated, and the results were expressed as mean ± standard deviation. Error bars representing standard deviation were included in the figures where applicable. For comparisons involving only two groups under the same experimental condition, statistical differences were evaluated using Student’s *t*-test. However, Student’s *t*-test was not applied to multi-point datasets such as pH-dependent activity, thermal stability, storage stability, and reusability assays. These datasets were presented descriptively as mean ± standard deviation unless an appropriate multiple-comparison analysis was performed. Therefore, pairwise statistical significance was not assigned to multi-point datasets without multiple-comparison analysis. A value of *p* < 0.05 was considered statistically significant where statistical testing was applied.

### 2.12. Use of Generative Artificial Intelligence Tool

During the preparation of this manuscript, ChatGPT by OpenAI (https://chatgpt.com, accessed on 10 August 2026) was used for language editing, grammar correction, academic wording improvement, and organization of the response to reviewers. The GenAI tool was not used to generate experimental data, perform data analysis, create raw results, interpret instrumental data, or draw scientific conclusions. All AI-assisted text was carefully reviewed, edited, and verified by the author, who takes full responsibility for the accuracy, integrity, and final content of the manuscript.

## 3. Results and Discussion

Enzyme immobilization is widely used to improve enzyme handling, recovery, and potential reuse in biotechnological applications. Different immobilization approaches, including adsorption, entrapment, ionic interaction, and covalent attachment, have been reported [27,31]. However, the performance of an immobilized enzyme system strongly depends on both the support material and the immobilization method. While strong enzyme–support interactions may improve enzyme retention, excessive chemical modification or unfavorable enzyme orientation may reduce catalytic activity by affecting enzyme conformation or active-site accessibility [32]. In the present study, AChE was directly immobilized onto the synthesized magnetic polymeric microspheres without using an external cross-linking agent. This cross-linker-free approach was selected to avoid possible activity loss associated with additional chemical coupling steps. The immobilization mechanism was mainly attributed to physical adsorption and multipoint non-covalent enzyme–support interactions. The phosphonic acid groups introduced by VPA, together with hydroxyl-containing TiO_2_ and Fe_3_O_4_ components, may contribute to hydrogen bonding, electrostatic interactions, and possible coordination-type interactions with functional groups of AChE molecules. These interactions may facilitate enzyme attachment onto the microsphere surface. However, because the system is mainly based on physical adsorption and non-covalent interactions, enzyme retention during long-term storage and repeated use may be limited. Therefore, the following results are discussed by considering both the advantages and limitations of this immobilization strategy.

### 3.1. Structural and Morphological Characterization

**Figure 1:**
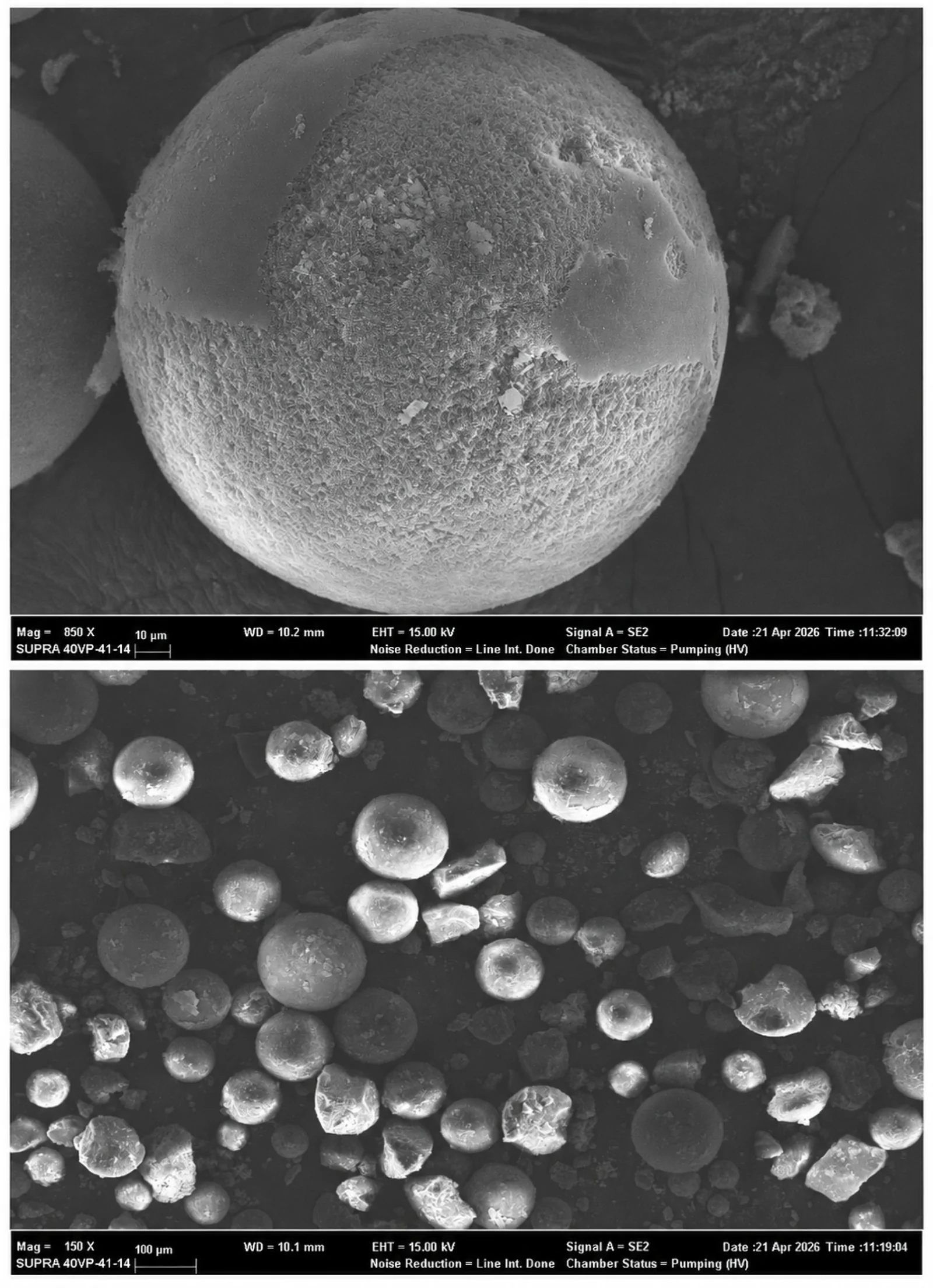
SEM images of m-poly(VPA-co-EGDMA)-TiO_2_ microspheres.

The morphological and structural properties of the synthesized microspheres were investigated using several characterization techniques. Figure 1 shows the SEM images of the microspheres. The white–gray appearance of the particles is attributed to the incorporation of magnetite and TiO_2_ nanoparticles within the polymer matrix. SEM images confirmed that the microspheres possessed a spherical morphology with a rough and porous surface. This porous morphology is advantageous for enzyme immobilization because it increases the available surface area and provides more interaction sites for enzyme attachment [33]. Minimal aggregation between microspheres was observed, indicating the homogeneous nature of the polymerization process. The particle size distribution determined by standard sieving ranged between 53 and 212 μm. The activity-based apparent immobilization yield of AChE onto the synthesized magnetic polymeric microspheres was calculated as 68%, supporting enzyme attachment onto the support material. This value should be interpreted as an activity-based immobilization parameter rather than as a direct protein-based enzyme-loading value. In contrast to glutaraldehyde-mediated covalent immobilization systems reported in the literature, the present system did not require an external cross-linking agent. Instead, AChE immobilization was mainly attributed to physical adsorption and multipoint non-covalent interactions between the enzyme and the functionalized microsphere surface. The phosphonic acid groups introduced by VPA, together with hydroxyl-containing TiO_2_ and Fe_3_O_4_ components, may facilitate enzyme attachment through hydrogen bonding, electrostatic interactions, and possible coordination-type interactions. Therefore, the porous morphology and functional surface chemistry of the microspheres are considered to play an important role in the observed activity-based immobilization performance [34].

**Figure 2:**
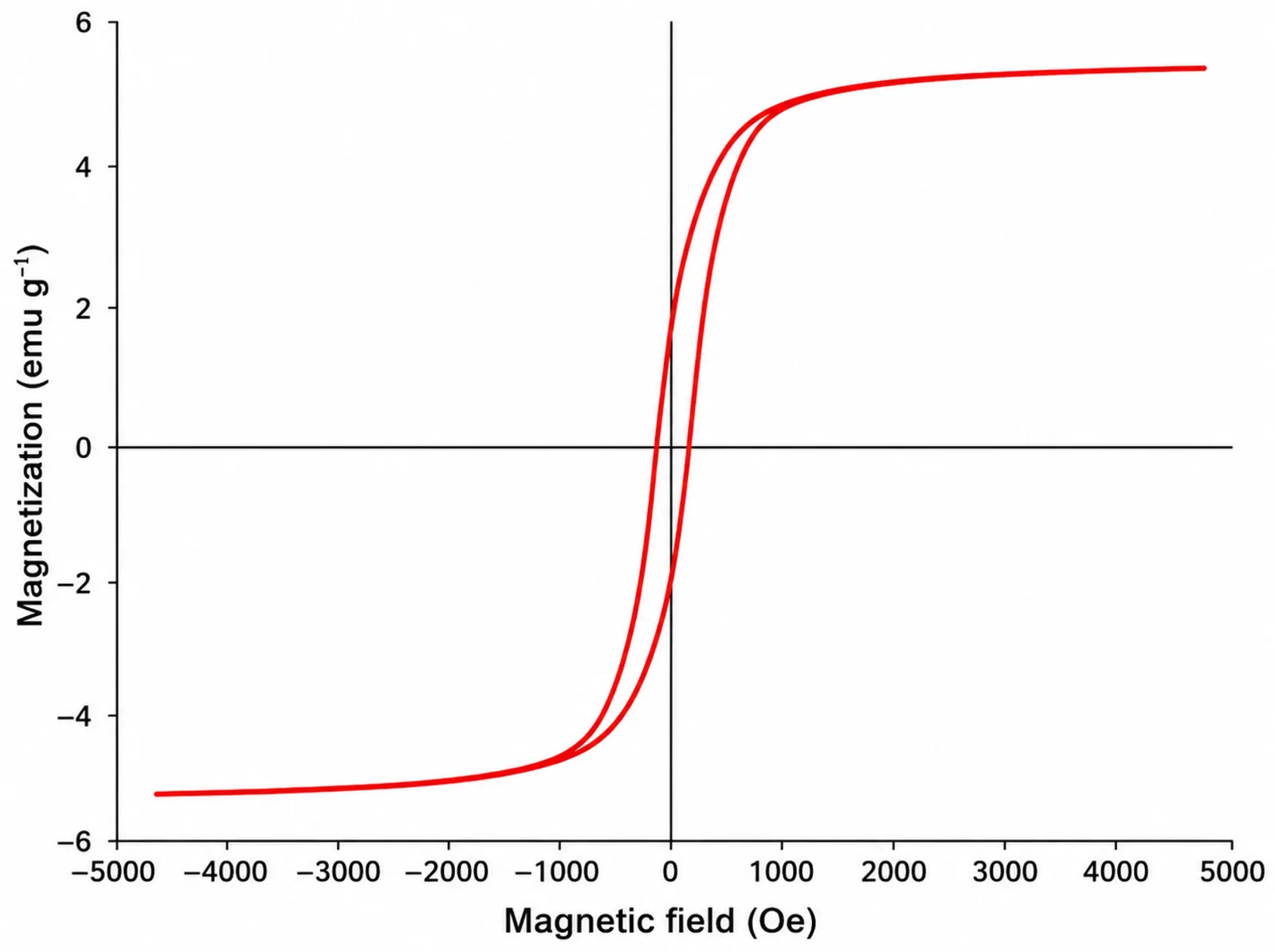
VSM magnetization curve of m-poly(VPA-co-EGDMA)-TiO_2_ magnetic microspheres.

The surface area and pore structure of the magnetic polymeric microspheres were evaluated by N_2_ adsorption–desorption analysis [35,36]. According to BET analysis, the specific surface area of the microspheres was determined to be 195 m^2^ g^−1^. This relatively high surface area can be attributed to the porous and cross-linked structure of the polymeric microspheres. The BJH pore size distribution indicated pore diameters ranging from 1.5 nm to 4.5 nm, corresponding to micro- and mesoporous regions. The presence of these pores is advantageous for enzyme immobilization because it increases the available surface area and provides additional interaction sites for AChE attachment. The increase in adsorption capacity at higher relative pressures may be associated with multilayer adsorption and capillary condensation within the mesoporous structure. The magnetic properties of the microspheres were evaluated using vibrating sample magnetometry (VSM), and the corresponding magnetization curve is presented in Figure 2. The density of the magnetic microspheres was measured as 1.43 g mL^−1^ at 25 °C, while the densities of Fe_3_O_4_ particles and the non-magnetic polymer matrix were 4.89 g mL^−1^ and 1.12 g mL^−1^, respectively. Based on these values, the volumetric fraction of Fe_3_O_4_ in the microspheres was calculated as approximately 8.2%, while the polymeric matrix constituted about 91.8% of the microsphere volume. The saturation magnetization of the microspheres was determined as 6.03 emu g^−1^. This relatively low magnetization value indicates weak-to-moderate magnetic responsiveness, which can be attributed to the low Fe_3_O_4_ content and the dominant non-magnetic polymeric phase. Nevertheless, the microspheres could be efficiently collected from aqueous suspension using an external magnet under the experimental conditions used in this study. This magnetic recoverability is an important feature for enzyme immobilization applications, as it enables facile separation and reuse of the AChE-loaded microspheres without requiring centrifugation or filtration.

**Figure 3:**
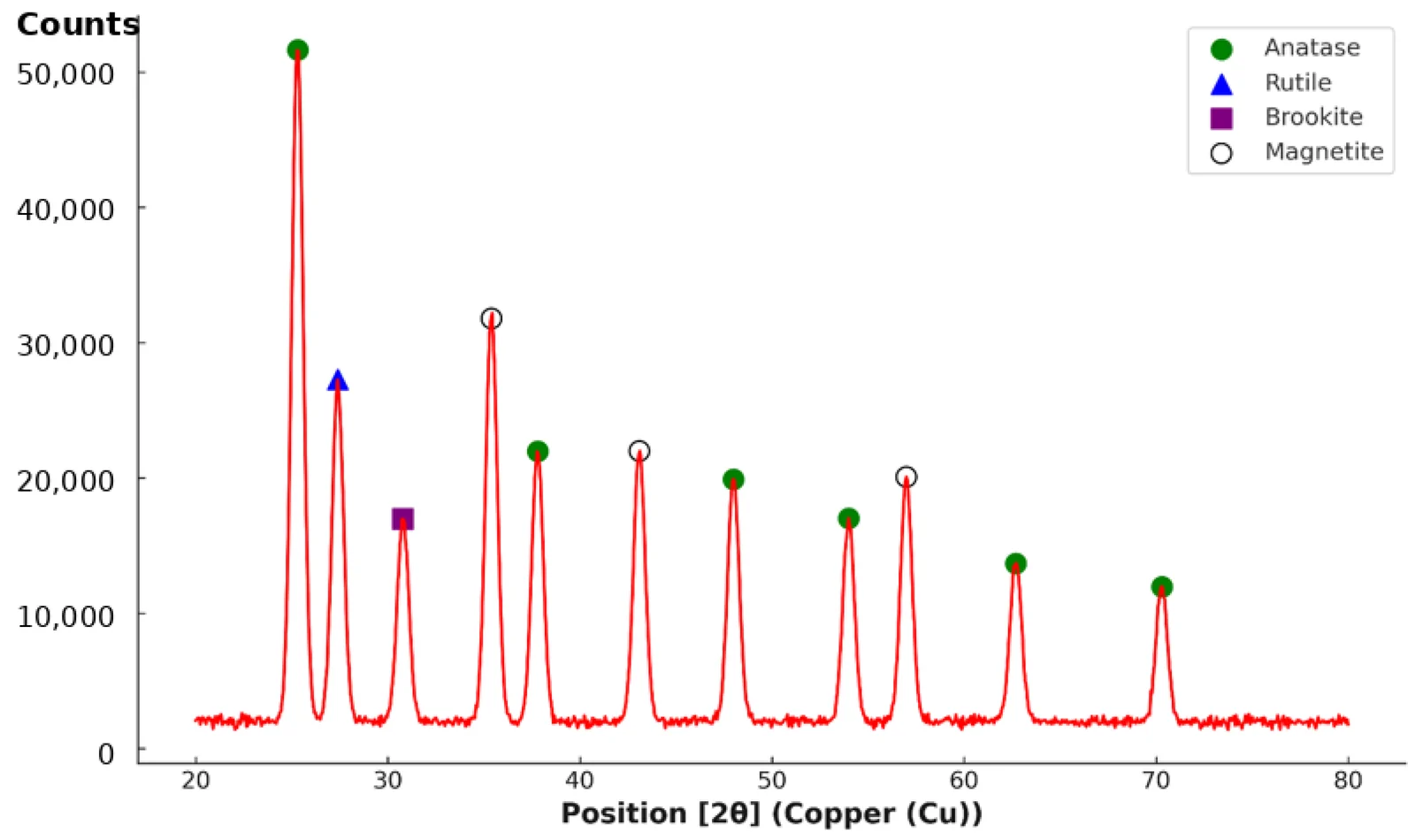
XRD pattern of m-poly(VPA-co-EGDMA)-TiO_2_ microspheres.

The crystalline structure of the synthesized microspheres was analyzed by X-ray diffraction (XRD) (Figure 3). The diffraction peaks were consistent with the presence of TiO_2_ and Fe_3_O_4_ phases within the polymeric microsphere structure. Additionally, diffraction peaks corresponding to Fe_3_O_4_ matched well with JCPDS standard card number 19-0629 for magnetite, Fe_3_O_4_, published by the Joint Committee on Powder Diffraction Standards/International Centre for Diffraction Data, Newtown Square, PA, USA. This finding confirms the face-centered cubic structure of magnetite. The broad background observed in the pattern indicates the amorphous nature of the polymer matrix.

**Figure 4:**
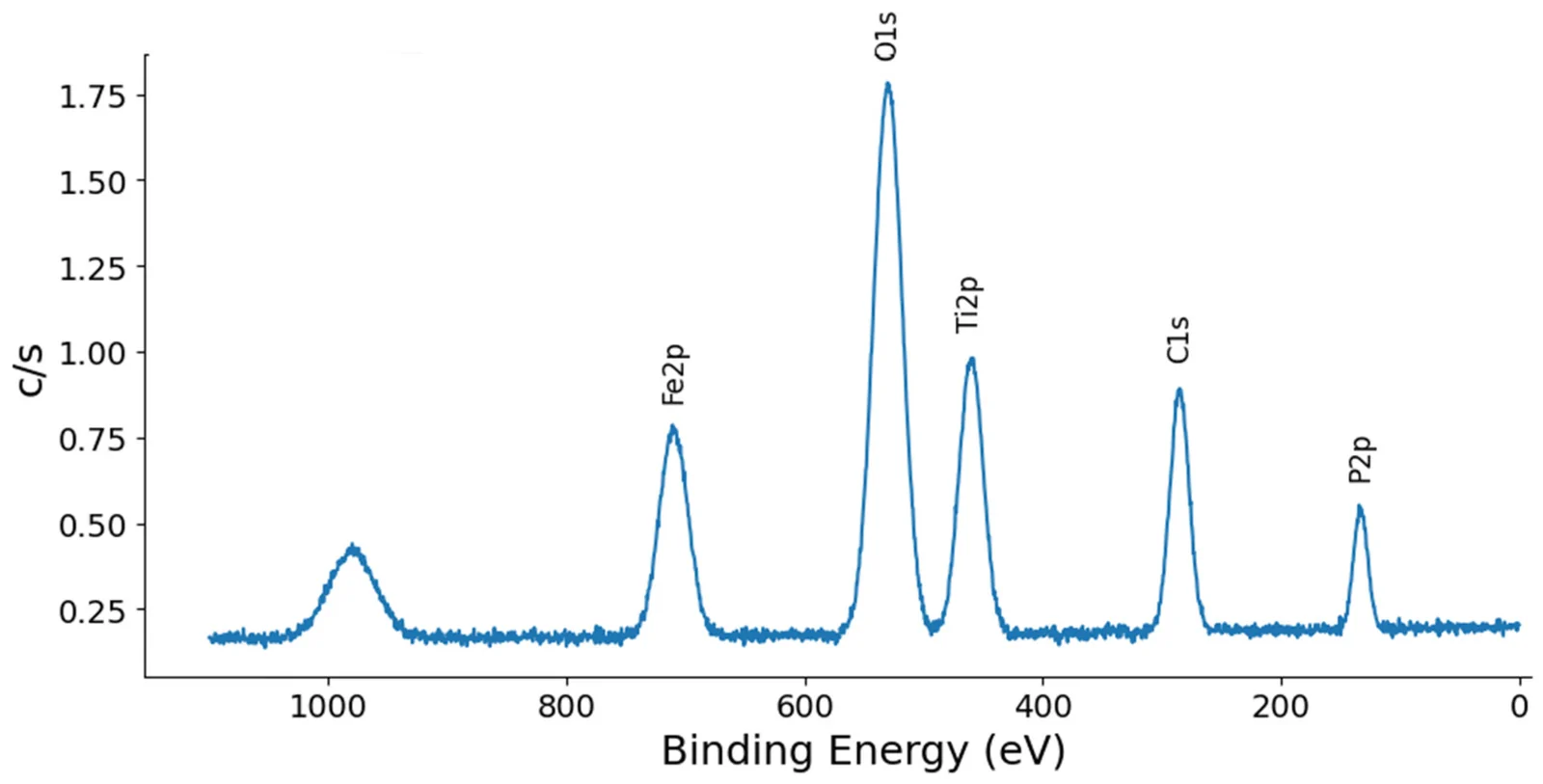
XPS spectrum of m-poly(VPA-co-EGDMA)-TiO_2_ microspheres.

Surface chemical composition was further analyzed by X-ray photoelectron spectroscopy (XPS) (Figure 4). The XPS results supported the presence of C, O, P, Ti, and Fe on the microsphere surface, consistent with the proposed composite structure. The relatively high carbon content can be attributed to the porous polymer matrix and minor surface contamination.

**Figure 5:**
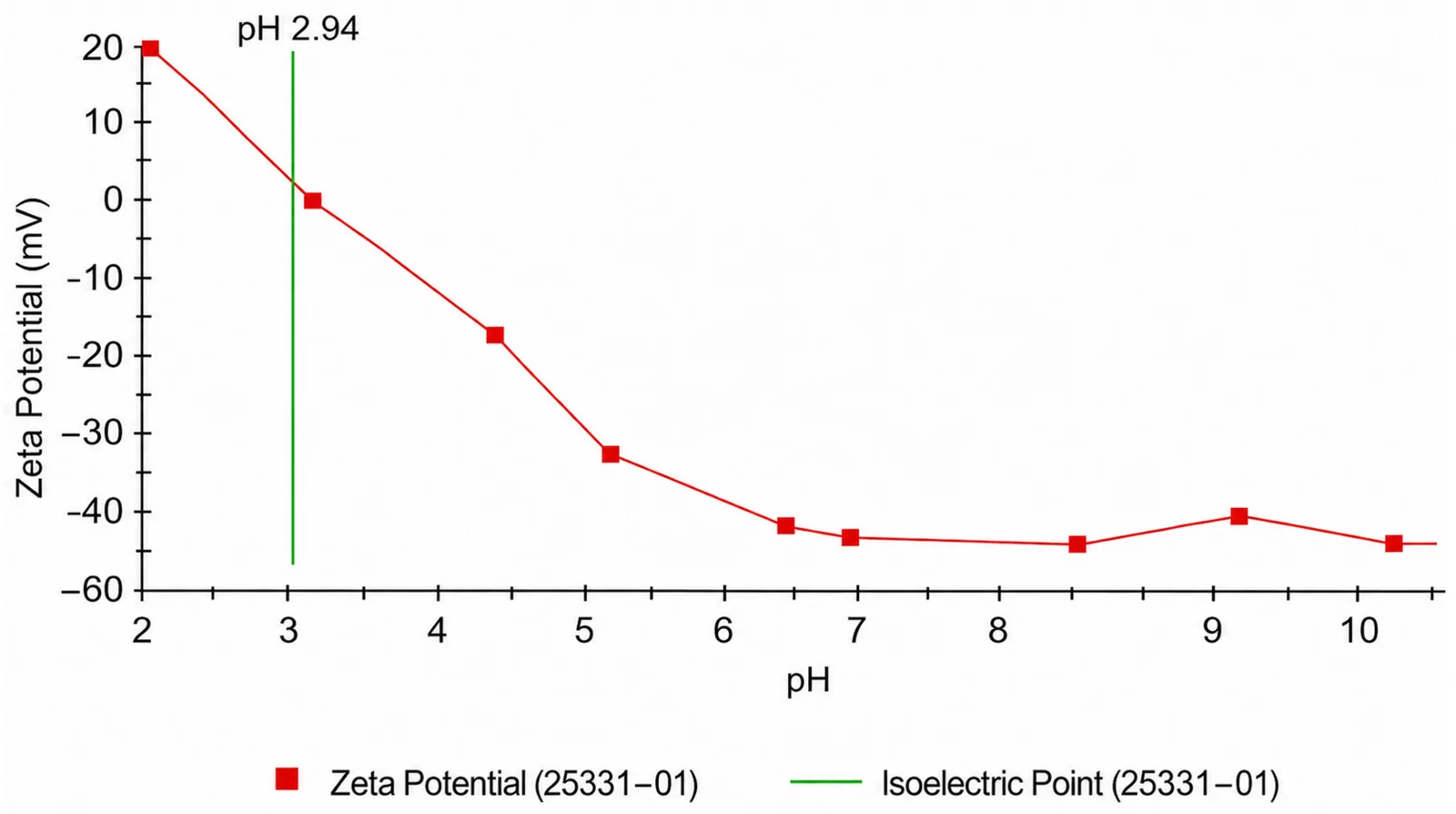
TGA thermogram of m-poly(VPA-co-EGDMA)-TiO_2_ microspheres.

Zeta potential analysis was conducted to determine the surface charge characteristics of the microspheres (Figure 5). The isoelectric point of the microspheres was determined to be approximately pH 3. Below this value, the microspheres possess a positive surface charge, whereas above this pH the surface charge becomes negative due to the ionization of phosphonic acid groups.

**Figure 6:**
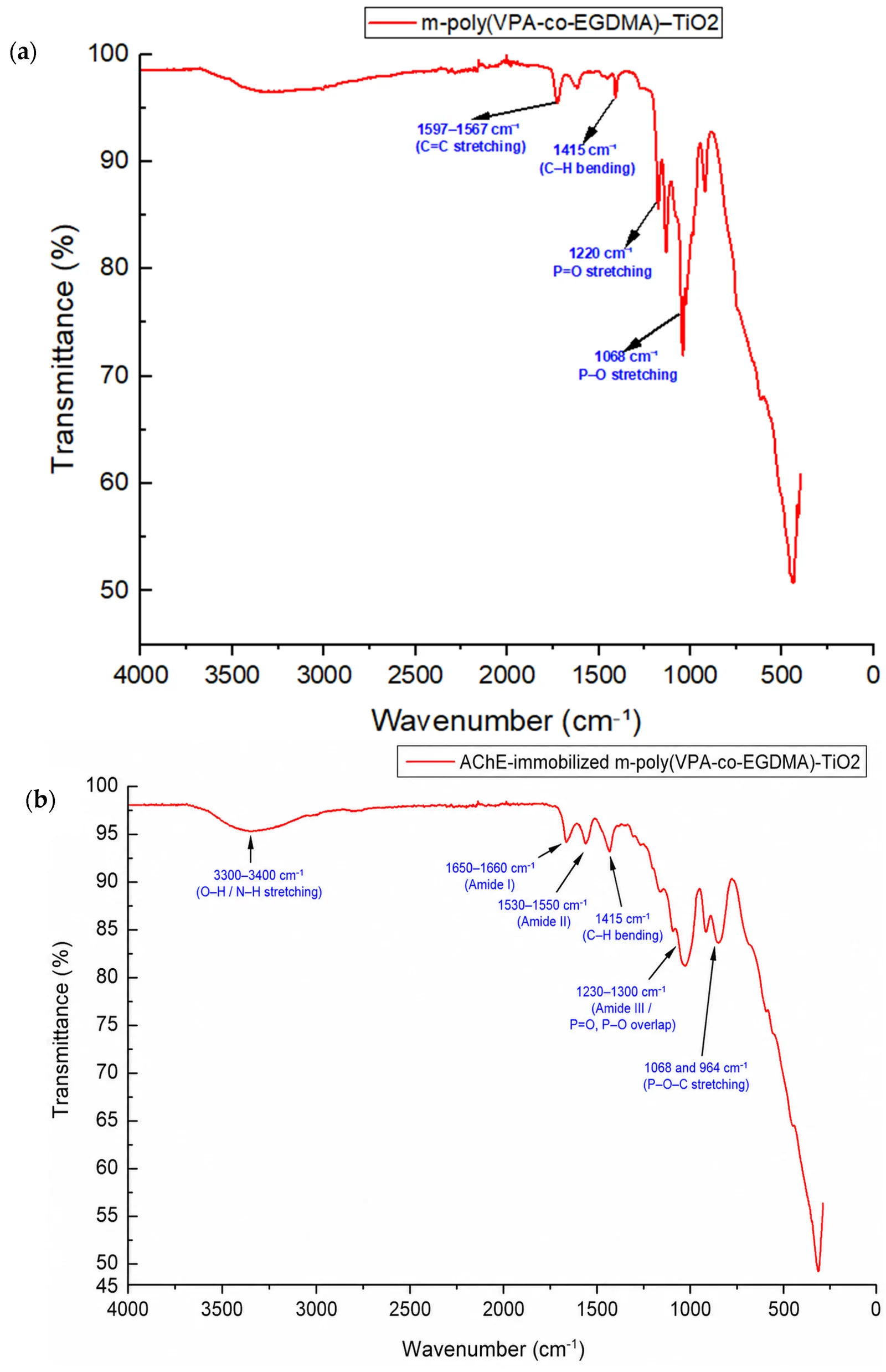
FT-IR spectra of microspheres: (**a**) synthesized m-poly(VPA-co-EGDMA)-TiO_2_ microspheres and (**b**) AChE-immobilized m-poly(VPA-co-EGDMA)-TiO_2_ microspheres.

The FT-IR spectrum of the synthesized microspheres exhibited characteristic absorption bands associated with the polymeric network and functional groups of VPA and EGDMA. Bands appearing in the 1597–1567 cm^−1^ region may be associated with weak C=C-related vibrations and/or overlapping contributions from the polymeric structure. The band at approximately 1415 cm^−1^ was assigned to C–H bending vibrations. The strong absorption band observed at approximately 1220 cm^−1^ was assigned to P=O stretching, confirming the presence of phosphonic acid groups derived from VPA. Additional peaks at 1068 and 964 cm^−1^ were attributed to P–O–C- and P–O-related stretching vibrations, supporting the formation of the VPA-containing polymeric microspheres. The FT-IR spectrum of the synthesized microspheres is presented in Figure 6a. After AChE immobilization, spectral changes associated with the protein structure of the enzyme were evaluated cautiously because several bands of the polymeric support overlap with characteristic protein absorption regions. The broad absorption band around 3300–3400 cm^−1^ was attributed to overlapping O–H and N–H stretching vibrations. The band observed around 1650–1660 cm^−1^ may be associated with the amide I band of the protein backbone, mainly arising from C=O stretching vibrations of peptide bonds. The region around 1530–1550 cm^−1^ may include contributions from the amide II band, which is mainly associated with N–H bending and C–N stretching vibrations of the protein backbone. However, this band is also visible in the spectrum of the non-enzyme-loaded m-poly(VPA-co-EGDMA)-TiO_2_ microspheres, indicating that it overlaps with absorption bands originating from the polymeric support. Therefore, the 1530–1550 cm^−1^ region was not considered as an independent or definitive indicator of AChE immobilization. It should be emphasized that amide I and amide II bands are characteristic of the protein backbone and are commonly used as indicators of protein secondary structure. However, due to spectral overlap with the support material in the present system, FT-IR results were interpreted only as supportive evidence for AChE immobilization rather than as definitive proof of a specific bonding mechanism or protein secondary-structure change. The appearance or relative intensification of protein-related bands, together with enzyme activity measurements, supports the successful immobilization of AChE onto the microspheres. The FT-IR spectrum of the AChE-immobilized microspheres is presented in Figure 6b.

**Figure 7:**
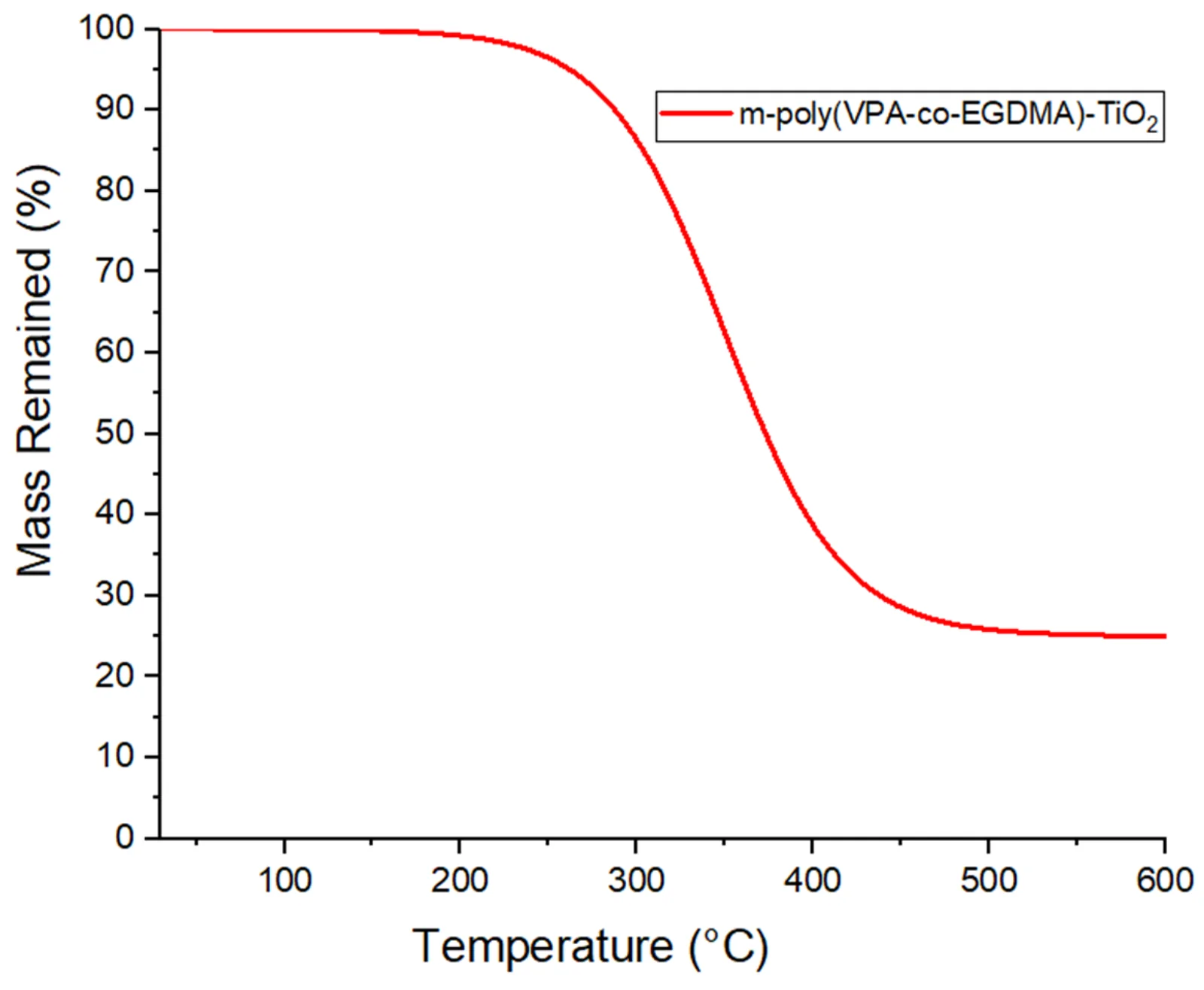
Thermogram of microspheres.

Thermal stability of the microspheres was evaluated by TGA/DTA analysis (Figure 7). An initial weight loss of approximately 8.72% below 200 °C was attributed to the removal of adsorbed water. The main thermal degradation of the polymer matrix occurred between 250 and 400 °C. A total weight loss of approximately 71.25% was observed, while the remaining 30% corresponded to the inorganic components TiO_2_ and Fe_3_O_4_. Above 500 °C, the mass remained nearly constant, indicating the stability of the inorganic oxides.

The magnetic particle volume fraction of the microspheres was calculated as approximately 8.2% based on the density values of the magnetic microspheres, Fe_3_O_4_ particles, and the non-magnetic polymer matrix. This result indicates that the microspheres contained a relatively low amount of magnetic inorganic phase, which is consistent with the weak-to-moderate magnetic responsiveness observed in the VSM analysis. The equilibrium swelling ratio of the microspheres was determined as 40.19%. This swelling behavior can be attributed to the porous and hydrophilic structure of the polymeric microspheres. The observed swelling capacity may facilitate the diffusion of aqueous media into the polymer network and provide accessible regions for enzyme attachment.

### 3.2. Immobilization of AChE on Polymeric Microspheres 

**Figure 8:**
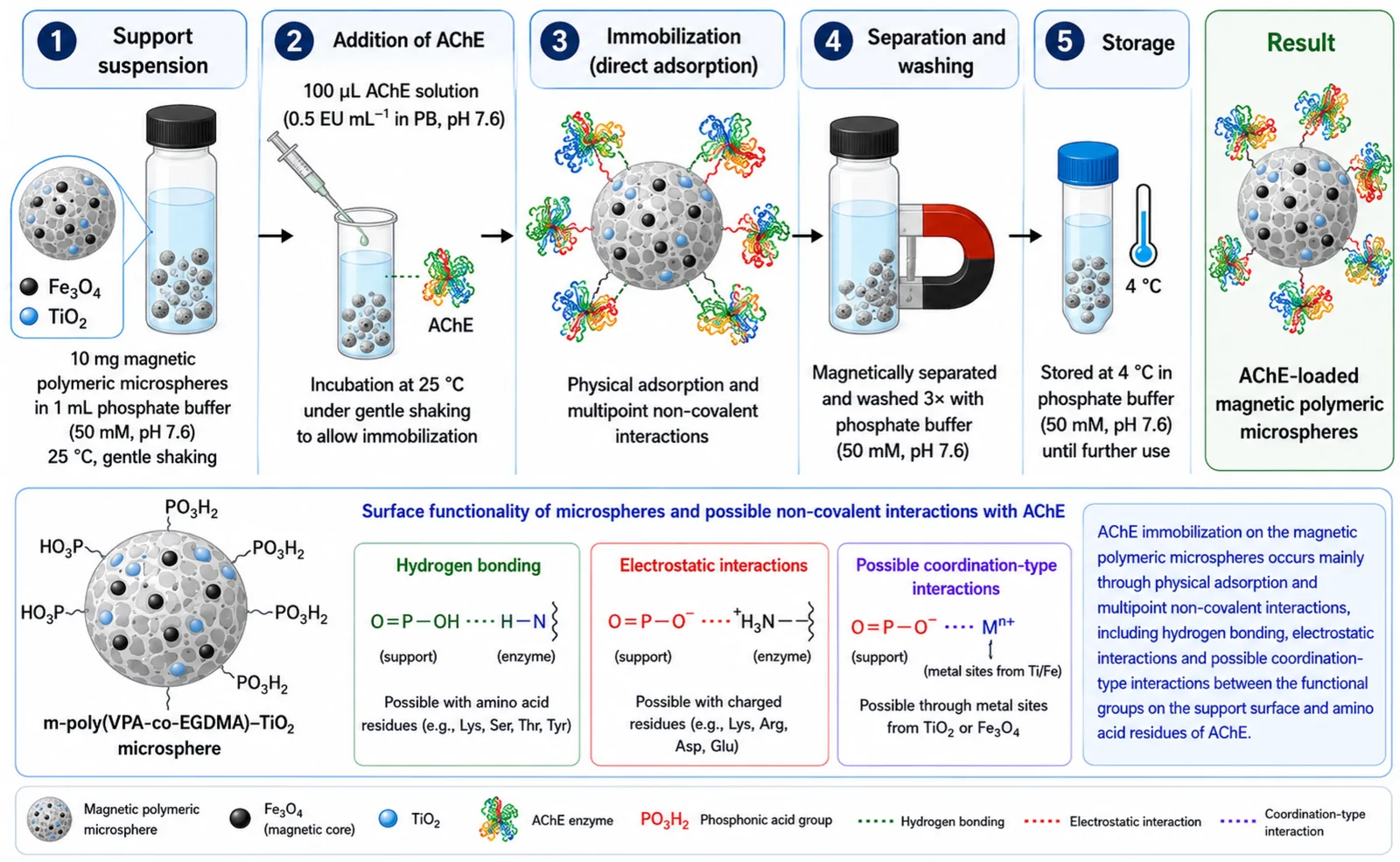
Schematic representation of AChE immobilization on magnetic m-poly(VPA-co-EGDMA)-TiO_2_ polymeric microspheres.

The activity-based apparent immobilization value of AChE on the functionalized magnetic polymeric microspheres was calculated as 68%. This value reflects the decrease in enzyme activity measured in the supernatant after immobilization and should not be interpreted as a complete immobilization yield, direct protein-based binding yield, enzyme loading, or activity recovery. A decrease in supernatant activity may result not only from enzyme attachment to the support but also from possible partial enzyme deactivation during the immobilization process. Therefore, this parameter alone cannot distinguish enzyme binding from activity loss [37,38]. The proposed immobilization mechanism is schematically shown in Figure 8. The results suggest that AChE immobilization occurred mainly through physical adsorption and multipoint non-covalent enzyme–support interactions. Phosphonic acid groups from VPA and hydroxyl-containing TiO_2_/Fe_3_O_4_ components may contribute to hydrogen bonding, electrostatic interactions, and possible coordination-type interactions with functional groups of AChE.

**Figure 9:**
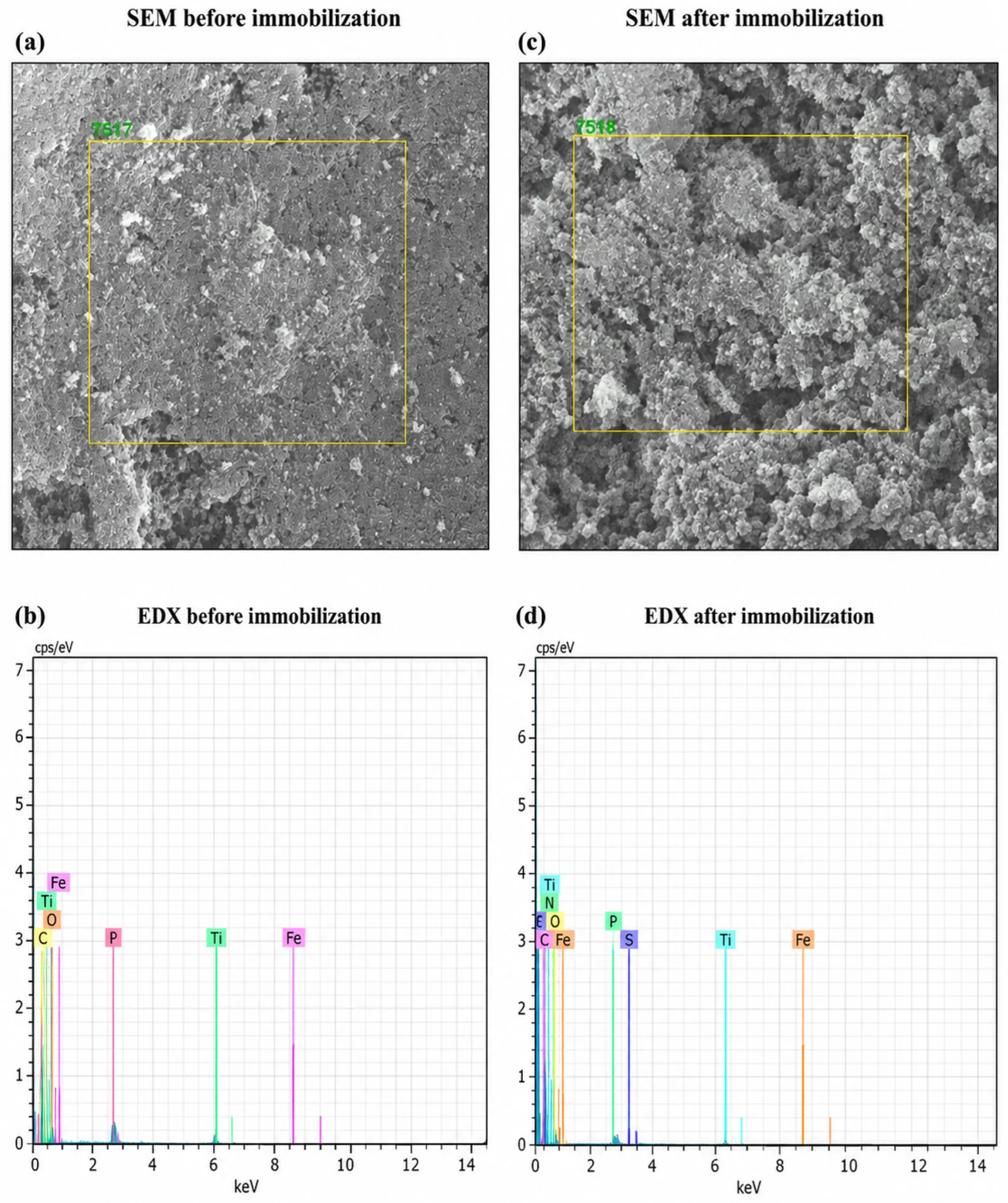
SEM–EDX analysis of (**a**,**b**) polymeric microspheres and (**c**,**d**) AChE-immobilized polymeric microspheres.

SEM–EDX analysis before and after immobilization is presented in Figure 9. The bare and AChE-loaded microspheres exhibited porous and heterogeneous surface morphologies. After immobilization, additional N and S signals were detected in the EDX spectrum, which may be associated with the protein structure of AChE. However, these SEM–EDX observations should be considered only as supportive and indirect evidence, because quantitative elemental composition and elemental mapping were not performed. Therefore, the immobilization results were interpreted together with the activity-based apparent immobilization value and FT-IR findings.

### 3.3. Functionalized Magnetic Polymeric Microspheres for AChE Immobilization: Kinetic Behavior, Stability, and Reusability 

The apparent kinetic parameters of free and immobilized AChE are summarized in Table 1. The apparent Km values of free and immobilized AChE were calculated as 0.020 ± 0.001 mM and 0.028 ± 0.002 mM, respectively. The Km values are reported in mM to ensure consistency with the substrate concentration range used in the kinetic experiments. The slight increase in apparent Km after immobilization suggests that substrate affinity may have been moderately affected by immobilization. Similar increases in Km have been reported for several immobilized AChE systems, particularly when enzyme molecules are attached to porous, polymeric, silica-based, or magnetic supports [39,40]. It should be emphasized that the reported Vmax values are expressed as EU.mL^−1^ and therefore depend on the amount of active enzyme present in the assay. Since protein-based enzyme loading and activity recovery were not quantitatively determined in the present study, the Vmax values should not be interpreted as direct measures of intrinsic catalytic productivity or relative catalytic productivity. Therefore, the lower Vmax value observed for immobilized AChE is discussed only as a decrease in apparent maximum reaction rate under the specific assay conditions used in this study. Overall, the kinetic results indicate that immobilization caused a slight increase in apparent Km and a lower apparent Vmax. These changes may be associated with restricted substrate accessibility, steric hindrance, unfavorable enzyme orientation, possible conformational changes, and possible mass-transfer limitations within the polymeric microsphere matrix. However, mass-transfer limitations were not directly confirmed experimentally and should therefore be considered only as a possible contributing factor [41].

### 3.4. pH-Dependent Activity of Free and Immobilized AChE

**Figure 10:**
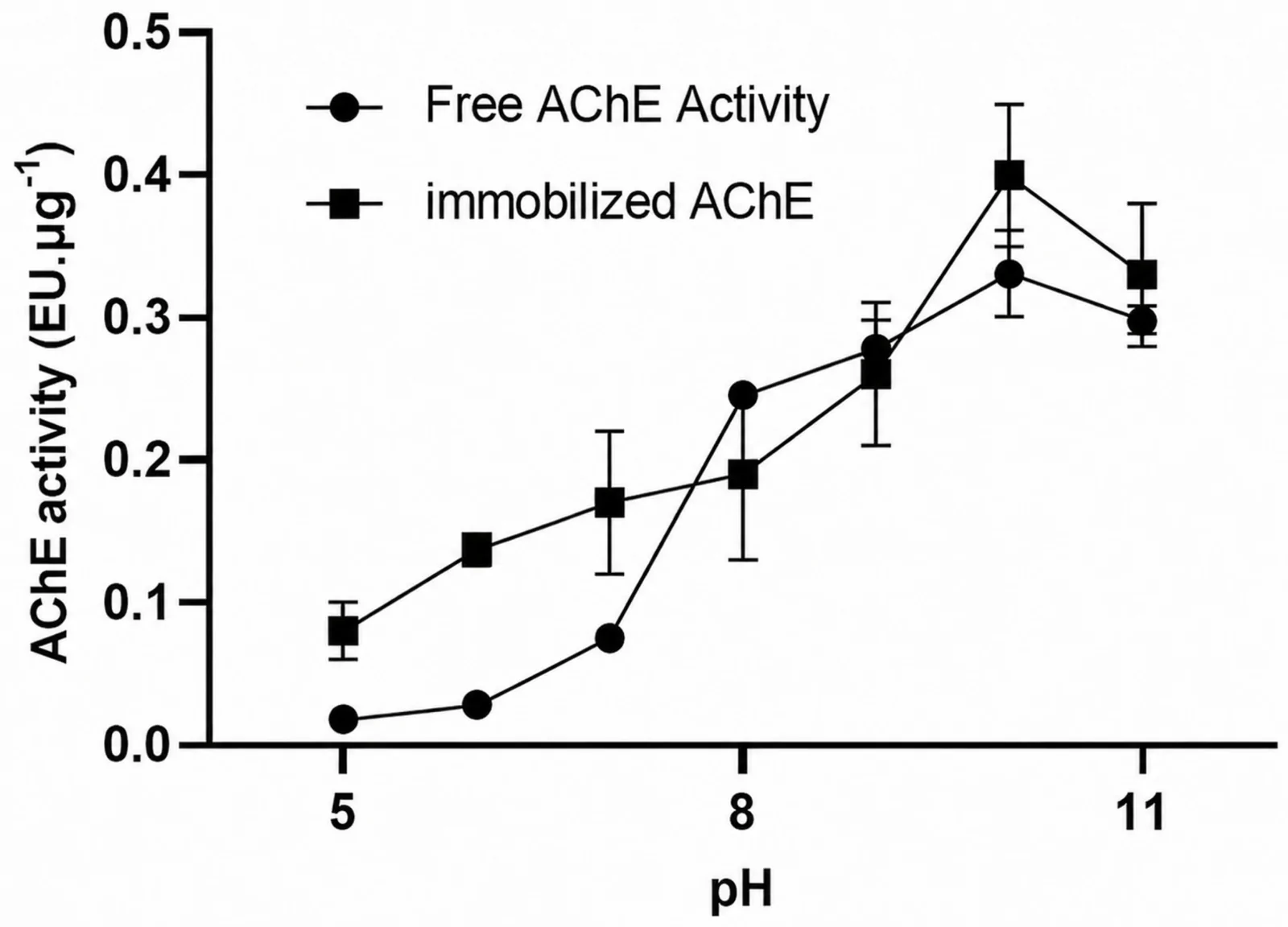
pH-dependent activity profiles of free and immobilized AChE. (Enzyme activity is expressed as AChE activity (EU.μg^−1^). Data are presented as mean ± standard deviation, *n* = 3.)

The pH-dependent activity profiles of free and immobilized AChE were evaluated between pH 5 and 11, and the results are presented in Figure 10. Enzyme activity is expressed as AChE activity (EU.μg^−1^) to ensure consistency with the activity unit stated in the Materials and Methods Section. Both free and immobilized AChE showed increased activity under alkaline conditions, with the highest measured activity observed at approximately pH 10. This result is consistent with several reported immobilized AChE systems in which immobilization altered the pH–activity profile or improved enzyme response under alkaline conditions [42,43]. In immobilized enzyme systems, such behavior is often attributed to the local microenvironment generated by the support surface, which may modify proton distribution, electrostatic interactions, or substrate accessibility around the enzyme. In the present system, the phosphonic acid groups introduced by VPA and the hydroxyl-containing TiO_2_/Fe_3_O_4_ components may contribute to the local charge environment around immobilized AChE. These functional groups may interact with amino-, carboxyl-, and hydroxyl-containing residues of the enzyme through hydrogen bonding, electrostatic interactions, and possible coordination-type interactions. Such multipoint non-covalent interactions may help partially preserve enzyme conformation under alkaline assay conditions. However, the improved activity observed at some pH values should not be interpreted as definitive evidence of improved pH stability, because the enzymes were not preincubated at different pH values followed by residual activity measurement under standard conditions. Therefore, the present results are discussed only as pH-dependent activity data. A decrease in activity was observed at pH 11 for both free and immobilized AChE, indicating that strongly alkaline conditions still adversely affect the enzyme. This suggests that although the microsphere support may provide a favorable local microenvironment within a certain pH range, it does not fully prevent alkaline inactivation. Compared with covalent or entrapment-based AChE immobilization systems, where stronger enzyme confinement may sometimes provide broader pH tolerance, the present adsorption-based system appears to provide only partial protection [44].

### 3.5. Storage Stability of Free and Immobilized AChE

**Figure 11:**
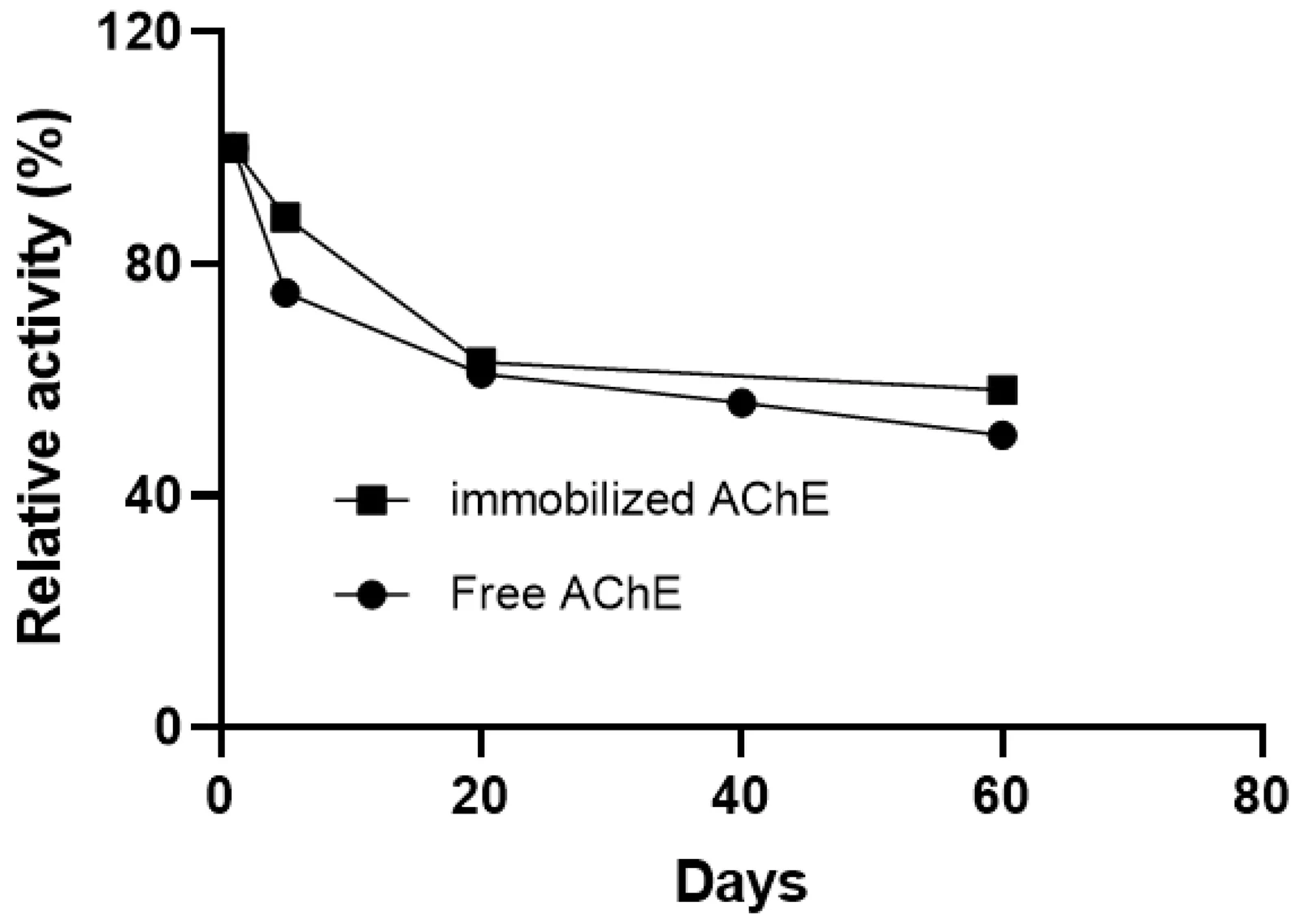
Storage stability profiles of free and immobilized AChE in Tris–HCl buffer (20 mM, pH 7.5) during the storage period.

The storage stability of free and immobilized AChE was examined in Tris/HCl buffer (20 mM, pH 7.5), and the results are presented in Figure 11. After 60 days of storage at 4 °C, immobilized AChE retained 58.2% of its initial activity, whereas free AChE retained 50.3%. This result indicates that immobilization produced only a moderate improvement in storage stability. Several published AChE immobilization systems, including silica-based supports, sol–gel matrices, nanocomposite supports, and covalently modified materials, have reported improved storage stability compared with free AChE [45,46,47]. In many of these systems, stronger enzyme–support interactions or physical confinement may reduce enzyme unfolding, aggregation, or autolysis during storage. In contrast, the limited improvement observed in the present study may be related to the immobilization mechanism. AChE was immobilized mainly through physical adsorption and multipoint non-covalent interactions rather than covalent bonding. Therefore, partial enzyme desorption or leakage into the storage medium may occur over time, reducing the long-term stabilizing effect of immobilization. This behavior is consistent with the known limitation of adsorption-based immobilization systems, in which mild immobilization conditions help preserve enzyme structure but weaker attachment may limit long-term retention. Although immobilized AChE showed slightly higher residual activity than the free enzyme, this difference should not be overinterpreted, particularly in the absence of detailed error analysis and statistical comparison for all storage points. Therefore, the storage stability results suggest only a limited stabilizing effect under the tested conditions. Nevertheless, the immobilized system provides a practical handling advantage because it can be magnetically recovered and separated from the reaction medium, whereas free AChE cannot be recovered in the same manner.

### 3.6. Reusability of Immobilized AChE

**Figure 12:**
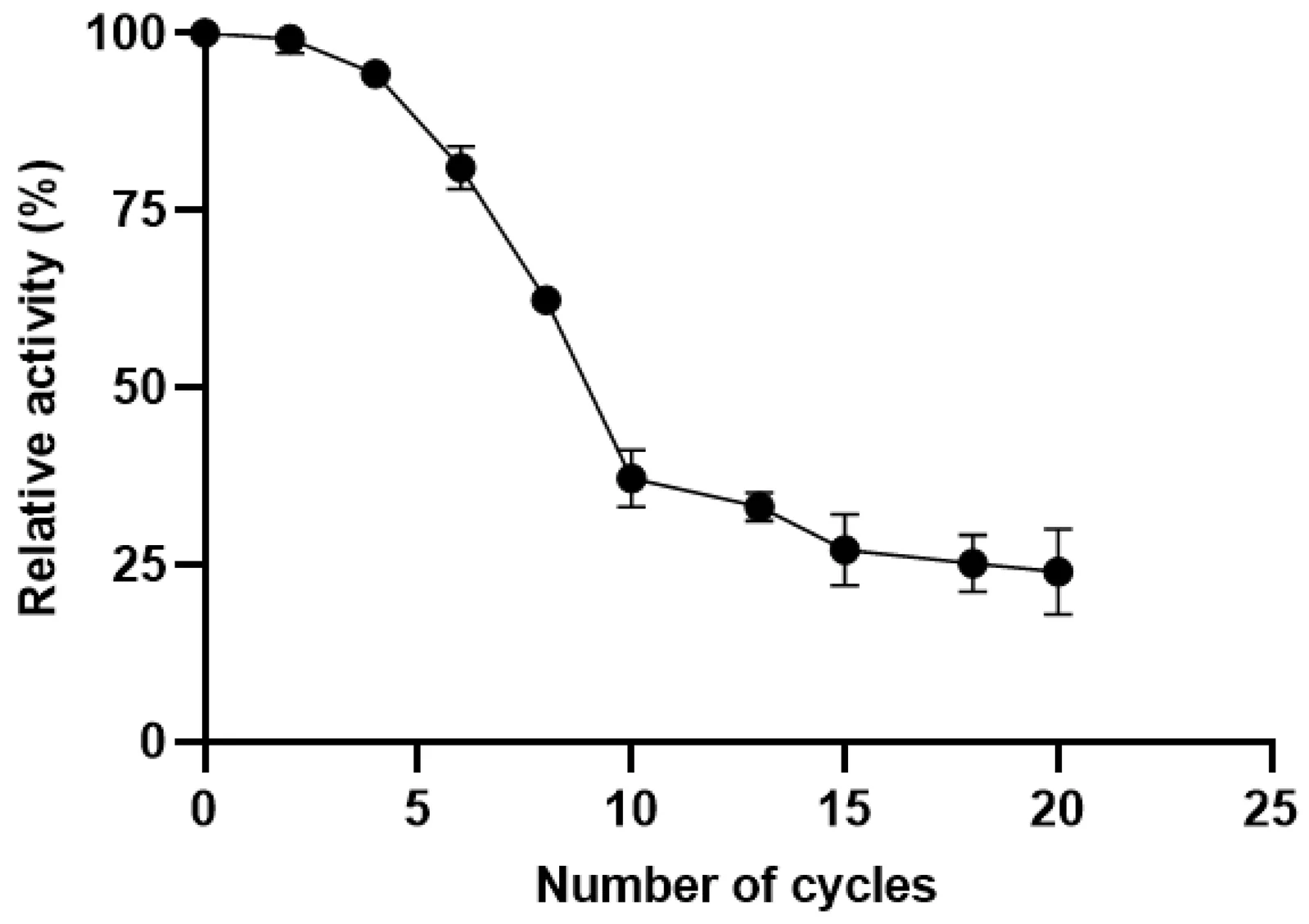
Reusability of immobilized AChE on microspheres.

The operational reusability of immobilized AChE was evaluated through repeated catalytic cycles, and the results are presented in Figure 12. Immobilized AChE retained relatively high activity during the first cycles; however, residual activity gradually decreased with repeated use and reached 24% after 20 cycles. This trend is consistent with many adsorption-based immobilized enzyme systems, where initial reuse performance may be acceptable but long-term operational stability is limited by enzyme leakage, weak enzyme–support interactions, mechanical loss of support particles, or gradual enzyme deactivation [48,49]. Compared with covalently immobilized or entrapped AChE systems, which may show improved resistance to enzyme leaching, the present cross-linker-free adsorption-based system provides a simpler and milder immobilization approach but may suffer from weaker enzyme retention during repeated washing and magnetic separation steps [48,50]. Therefore, the decline in residual activity after repeated cycles is likely associated with partial enzyme desorption, random enzyme orientation, possible conformational changes, and mechanical loss of microspheres during handling. The presence of Fe_3_O_4_ provides an important operational advantage by enabling magnetic recovery without centrifugation or filtration. Similar advantages have been reported for magnetic enzyme supports, where magnetic separation simplifies reuse and reduces processing time [51]. However, magnetic recoverability alone is not sufficient to ensure long-term operational durability. The present results therefore indicate that the microsphere system provides initial reusability, but further optimization is required to improve enzyme retention and long-term reuse performance.

### 3.7. Thermal Stability of Free and Immobilized AChE

**Figure 13:**
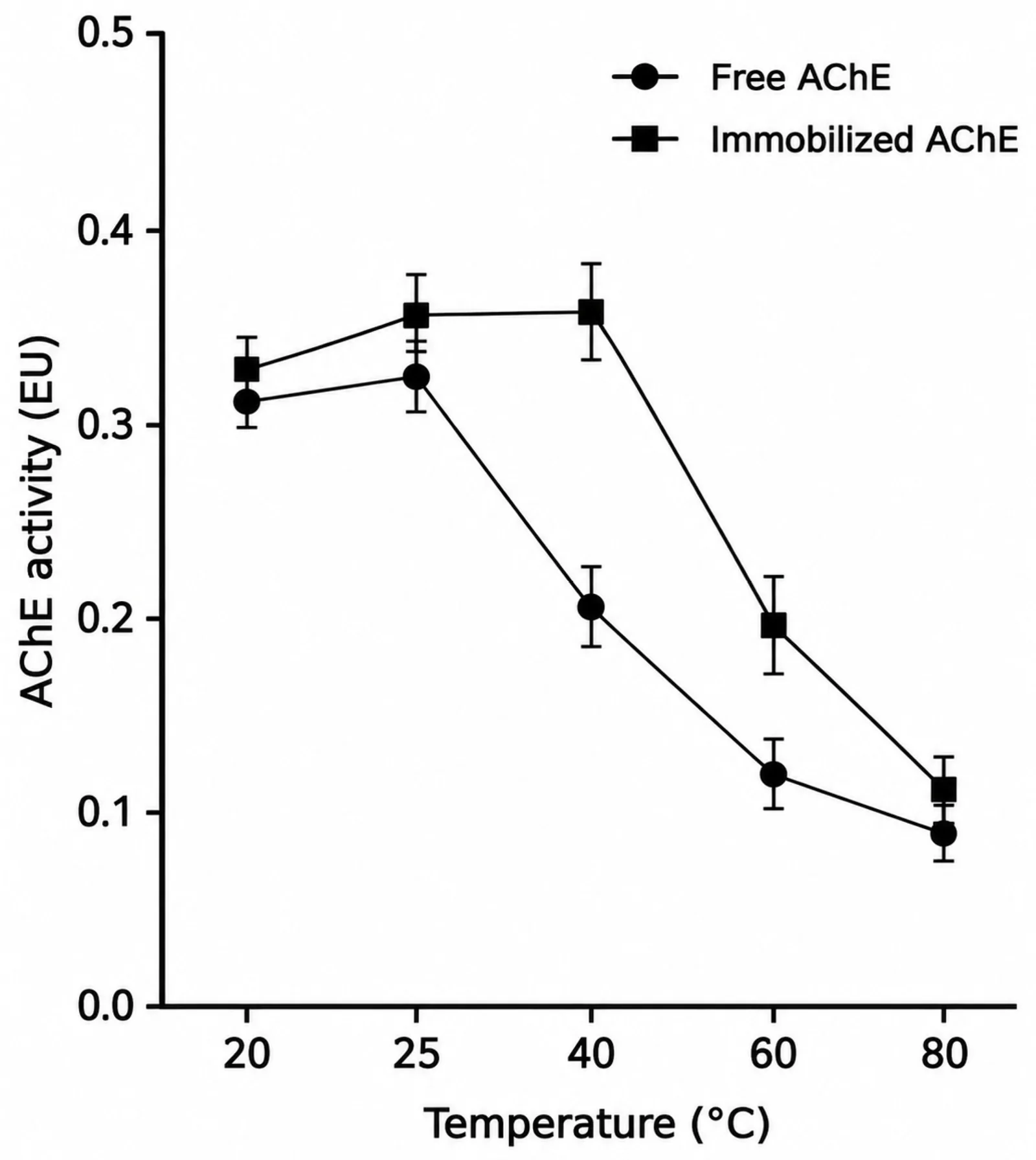
Thermal stability profiles of free and immobilized AChE after incubation at different temperatures for 2 h.

The thermal stability of free and immobilized AChE was evaluated after incubation at different temperatures between 20 and 80 °C for 2 h, and the results are presented in Figure 13. Since the activity measurements were performed after thermal incubation under standard assay conditions, these data represent residual activity after heat treatment rather than the direct effect of reaction temperature on catalytic activity. Therefore, the results should not be interpreted as an optimum catalytic temperature or as a temperature-dependent activity profile. After thermal incubation, immobilized AChE retained relatively higher residual activity than free AChE at elevated temperatures, particularly in the 40–60 °C range. Similar protective effects have been reported for immobilized AChE in alginate- and carrageenan-based matrices and other immobilized enzyme systems, where confinement within a support matrix reduces conformational flexibility and improves resistance to thermal unfolding [52]. In the present system, the relatively higher residual activity of immobilized AChE may be associated with multipoint non-covalent enzyme–support interactions involving phosphonic acid groups, TiO_2_/Fe_3_O_4_ components, and the porous polymeric matrix. However, residual activity decreased at higher temperatures for both free and immobilized AChE, indicating that immobilization did not fully prevent thermal inactivation. This suggests that the protective effect of the current support is modest. Stronger stabilization may require optimization of enzyme orientation, immobilization density, pore architecture, surface functionality, or the introduction of controlled covalent attachment strategies. Thus, the thermal stability results support the conclusion that the developed support provides partial thermal protection but requires further optimization for high-temperature or long-term operational applications.

### 3.8. Critical Evaluation and Comparison with Reported AChE Immobilization Systems

The novelty of this study lies in the development of magnetic m-poly(VPA-co-EGDMA)-TiO_2_ porous microspheres as multifunctional supports for direct AChE immobilization. Unlike many conventional immobilization systems that rely on either polymeric supports, silica-based matrices, sol–gel entrapment, or magnetic nanoparticles alone, the present system combines magnetic recoverability, porous morphology, phosphonic acid functionality, TiO_2_ incorporation, and a cross-linked polymeric network within a single microsphere architecture. This integrated structure provides functional sites for enzyme–support interactions, accessible regions for enzyme attachment, and magnetic responsiveness for easy separation from the reaction medium. As shown in Table 2, the apparent Km value obtained for immobilized AChE in this study (0.028 mM) is comparable to values reported for several immobilized AChE systems, although direct comparison should be made cautiously because of differences in enzyme source, substrate concentration, assay method, immobilization protocol, support type, data fitting procedure, and activity units. In addition, the lower apparent Vmax value of immobilized AChE compared with free AChE follows a common trend reported in the literature, where immobilization may reduce apparent maximum reaction rate because of diffusional and conformational restrictions while improving separation, handling, or reuse capability.

The storage stability and reusability results also show both advantages and limitations of the present system. Immobilized AChE retained slightly higher activity than free AChE after 60 days, but this improvement was moderate. Similarly, the immobilized enzyme showed useful initial reusability but substantial activity loss after repeated cycles. Compared with systems based on stronger covalent attachment, entrapment, or enzyme aggregates, the present adsorption-based system may offer simpler preparation and milder immobilization conditions, but weaker enzyme retention may limit long-term performance. Overall, magnetic m-poly(VPA-co-EGDMA)-TiO_2_ polymeric microspheres provide a promising but preliminary support for direct AChE immobilization. The main advantages of the system are magnetic recoverability, porous morphology, functional surface chemistry, and initial reusability. However, the lower apparent Vmax value, moderate storage-stability improvement, and gradual activity loss during repeated cycles indicate that further optimization is required before practical biosensing or environmental monitoring applications can be fully realized. Future studies should focus on quantitatively determining enzyme loading and activity recovery, optimizing enzyme orientation, improving pore structure and particle morphology, strengthening enzyme–support interactions, and evaluating the system using real inhibitor-detection or biosensing models.

## 4. Conclusions

In this study, magnetic m-poly(VPA-co-EGDMA)-TiO_2_ porous microspheres were prepared via suspension polymerization and evaluated as supports for direct AChE immobilization. The support preparation was based on a polymeric microsphere synthesis approach adapted from previously reported methods, while the present work focused on its application and evaluation for AChE immobilization. The microspheres exhibited porous morphology, magnetic recoverability, and functional surface characteristics that may provide accessible regions and interaction sites for enzyme attachment. AChE was immobilized mainly through physical adsorption and multipoint non-covalent enzyme–support interactions without using an external cross-linking agent. Immobilization caused a slight increase in apparent Km and a lower apparent Vmax under the tested assay conditions. Since protein-based enzyme loading and activity recovery were not quantitatively determined, the Vmax values were interpreted only as apparent maximum reaction rates rather than as measures of intrinsic or relative catalytic productivity. The immobilized enzyme showed moderate storage-stability improvement and initial reusability; however, the gradual decrease in residual activity during repeated cycles indicates limited long-term operational durability. The pH-dependent activity, thermal stability, storage stability, and reusability results suggest that the microsphere platform offers practical advantages in terms of magnetic recovery and enzyme handling. However, the current system should be considered a preliminary immobilization platform rather than a fully optimized biocatalyst. Future studies should focus on quantitative enzyme loading, activity recovery, enzyme orientation, pore structure, particle morphology, and strengthened enzyme–support interactions to improve apparent kinetic performance and long-term operational stability.

## Figures and Tables

**Table 1:** The obtained kinetic parameters are summarized.

Enzyme Sample	Km (mM)	Vmax (EU.mL^−1^)
Free AChE	0.020 ± 0.001	0.502 ± 0.073
Immobilized AChE	0.028 ± 0.002	0.132 ± 0.053

**Table 2:** Quantitative Comparison of AChE Immobilization Systems.

Support Material/System	Km (mM)	Vmax (EU.mL^−1^)	Storage Stability	Reusability	Reference
Free AChE (This study)	0.020 ± 0.01	0.502 ± 0.07	50.3% after 60 days at 4 °C	—	This study
m-poly(VPA-co-EGDMA)-TiO_2_ (This study)	0.028 ± 0.01	0.132 ± 0.05	58.2% after 60 days at 4 °C	24% after 20 cycles	This study
Polyacrylic acid nanofiber membrane	0.031–0.045	~0.10–0.20	~60–70%	Not reported	[7]
Mesoporous silica-based supports	0.030–0.060	~0.12–0.25	High (~65–80%)	Not reported	[11,12]
Polymer-based porous microspheres (GMA-EGDMA)	0.022–0.040	~0.10–0.22	Not reported	Not reported	[53]
Magnetic polymer nanocomposites	0.020–0.045	~0.12–0.26	Not reported	Not reported	[54,55]
Silica–enzyme aggregates (CLEAs)	0.025–0.050	~0.08–0.18	Not reported	Not reported	[12,56]

## Data Availability

The original contributions presented in this study are included in the article. Further inquiries can be directed to the corresponding author.

## Abbreviations

The following abbreviations are used in this manuscript:

AChE	Acetylcholinesterase
ATChI	Acetylthiocholine iodide
BET	Brunauer–Emmett–Teller
BJH	Barrett–Joyner–Halenda
BPO	Benzoyl peroxide
EGDMA	Ethylene glycol dimethacrylate
VSM	Vibrating sample magnetometry
FT-IR	Fourier transform infrared spectroscopy
MPMs	Magnetic polymeric microspheres
PVA	Poly(vinyl alcohol)
SEM	Scanning electron microscopy
EDX	Energy dispersive X-ray spectroscopy
TGA	Thermogravimetric analysis
DTA	Differential thermal analysis
VPA	Vinylphosphonic acid
XPS	X-ray photoelectron spectroscopy
XRD	X-ray diffraction
